# Prevalence of high-risk human papillomavirus and abnormal pap smears in female sex workers compared to the general population in Antwerp, Belgium

**DOI:** 10.1186/s12889-016-3099-5

**Published:** 2016-06-07

**Authors:** Alex Vorsters, Tine Cornelissen, Elke Leuridan, Johannes Bogers, Davy Vanden Broeck, Ina Benoy, Herman Goossens, Niel Hens, Pierre Van Damme

**Affiliations:** Centre for the Evaluation of Vaccination (CEV), Vaccine and Infectious Disease Institute (VAXINFECTIO), University of Antwerp, Campus Drie Eiken, Universiteitsplein 1, B 2610 Antwerp, Belgium; Antwerp Health House for Prostitution, Ghapro vzw, Antwerp, Belgium; Algemeen Medisch Laboratorium (part of National Reference Centre for HPV), Emiel Vloorsstraat 9, 2020 Antwerp, Belgium; Applied Molecular Biology Research (AMBIOR), University of Antwerp, Antwerp, Belgium; International Centre for Reproductive Health (ICRH), Ghent University, Ghent, Belgium; Laboratory of Medical Microbiology, Vaccine and Infectious Disease Institute, University of Antwerp, Antwerp, Belgium; Clinical Microbiology, Antwerp University Hospital, Antwerp, Belgium; Interuniversity Institute for Biostatistics and Statistical Bioinformatics (I-BIOSTAT), Hasselt University, Hasselt, Belgium; Centre for Health Economics Research and Modeling Infectious Diseases (CHERMID), Vaccine and Infectious Disease Institute (VAXINFECTIO), University of Antwerp, Antwerp, Belgium; Epidemiology and Social Medicine, University of Antwerp, Antwerp, Belgium

**Keywords:** Human papilloma virus, Female sex workers, Epidemiology, Cervical cancer, Prevention

## Abstract

**Background:**

Although female sex workers (FSWs) are a well-known high-risk group for Human Papillomavirus (HPV) infections, few tailored intervention programmes for HPV have been established worldwide. The lack of reliable data on the prevalence of HPV and related cervical lesions hampers the establishment of evidence-based intervention programmes.

The objectives of this study were to describe the prevalence of high-risk Human Papillomavirus (hrHPV) infections and abnormal pap smears in FSWs compared to a control group in Antwerp, Belgium.

**Methods:**

HPV genotyping and cytology data were analysed from routine Pap smear tests that were collected from both FSWs and the general population (1334 samples for each group) between June 2006 and June 2010. Within the laboratory database, all FSWs were matched 1:1 for age and testing date to determine the ORs of hrHPV genotypes, DNA and cytology outcome.

**Results:**

The prevalence of hrHPV DNA in FSWs was 41.7 % compared to 19.8 % in the age-matched controls with an overall OR of 2.8 (95 % CI: 2.3–3.4). Significant differences were observed in all age groups, and the most significant differences were observed in the cohort under 21 years of age (prevalence of 64.4 % in FSWs versus 14.8 % in controls; OR 10.3 (95 % CI: 5.0–21.2). Significantly more cervical lesions were observed in FSWs, particularly in the 17- to 21-year old age group (OR for LSIL or HSIL: 10.3 (95 % CI: 3.2–33.8). In both groups, HPV 16 was the most prevalent at 12.1 and 6.6 % in the FSW and control groups, respectively. HPV 18 was the 8^th^ and 7^th^ most frequent genotype at 5.0 and 2.5 % in the FSW and control groups, respectively.

**Conclusions:**

FSWs have a significantly higher prevalence of hrHPV and more abnormal Pap smears than does the general population in Antwerp, Belgium. The hrHPV prevalence in FSWs is similar to that reported in the literature. The need for tailored intervention programmes should be investigated further.

## Background

Worldwide, cervical cancer is the fourth most frequent type of cancer in women and causes an estimated 270,000 deaths every year [1]. High-risk Human Papillomavirus (hrHPV) infections are encountered in 90–100 % of all cervical tumours [2–4]. However, over 70 % of hrHPV infections are transient [5]. Worldwide, the overall prevalence of HPV for women with normal cytology is 10 % [6, 7].

In Belgium, the most common hrHPV genotypes are type 16 (3.7 %), 31 (3.0 %), 51 (2.3 %), 53 (2.1 %) and 59 (1.7 %), followed by HPV type 18 (1.5 %) [8]. In general, well-known cofactors for HPV infection include low socioeconomic status, long-term use of oral contraception, sexually promiscuous behaviour, smoking, HSV-2 sero-positivity, and high parity [9].

Although female sex workers (FSWs) are a well-known high-risk group for HPV infections, few tailored intervention programmes for HPV have been established worldwide. The lack of reliable data on the prevalence of HPV and related cervical lesions hampers the establishment of evidence-based intervention programmes. In addition, a direct comparison with an age-matched control group is often difficult due to a lack of data, and the use of different laboratory tests makes comparisons among studies difficult. However, the major hurdle is that many FSWs start sex work when they are younger than the screening age recommended by the respective national cervical cancer screening programmes, and therefore, there often is no readily available control group among younger age cohorts.

The main aim of this cross-sectional survey is to determine the prevalence of hrHPV and HPV genotype distributions among FSWs in Antwerp. In addition, the OR for hrHPV DNA positivity, HPV genotypes and cytology compared to the general population are investigated.

## Methods

The study was performed in accordance with the Helsinki Declaration, procedures established by Belgian law and the guidelines of ICH-GCP. Ethical approval was obtained from the medical ethical board of Antwerp University (Ref: B30020072416).

### Setting and population

The Antwerp health house for prostitution (Ghapro) offers sex workers free and anonymous screenings for sexually transmitted infections (STIs) as well as advice and education on sex work-related issues (http://www.ghapro.be/en/index.html). This nested case-control study was based on the results of cervical cytological screenings and hrHPV testing of all FSWs visiting the Ghapro clinic between June 2006 and June 2010. This data was selected from the laboratory database of the Laboratory for Clinical and Molecular Pathology (part of National Reference Center for HPV), Antwerp, Belgium. All samples were processed in this laboratory. For women tested on multiple occasions during this period, only the first screening results were included.

A control group was selected from the same laboratory database (general female population). All FSWs were matched 1:1 for age, testing date, and location (Antwerp region). As with the FSWs, only the first set of results during this testing period was taken into account for the controls.

Pregnant women were not screened. Women with immunodeficiency conditions, such as HIV, and women without a reported age or date of birth were excluded from the study.

### Laboratory analysis

#### Sample processing and cytological procedure

Cervical cells were collected using the Cervix-Brush® Combi (Rovers, Oss, The Netherlands). After collection, brush heads were transferred directly into alcohol-based preservatives (BD SurePath™, BD Diagnostics – TriPath, NC, USA). All vials were transported to the Laboratory for Clinical and Molecular Pathology, Antwerp, Belgium. Thin-layer slides were prepared with the robotic BD PrepStain™ Slide Processor (BD Diagnostics–TriPath, Burlington, NC, USA) [10]. Cytology screening was performed with prior knowledge of HPV identically in both groups as described earlier [11]. The cytological results were reported according to the Bethesda system 2001 [12].

#### Type-specific HPV detection

DNA isolation from liquid-based cytology was performed as previously described [13]. A clinically validated, real-time, quantitative PCR was used to amplify 18 HPV types: HPV 6E6, 11E6, 16E7, 18E7, 31E6, 33E6, 35E6, 39E7, 45E7, 51E7, 52E7, 53E6, 56E7, 58E7, 59E7, 66E6, 67 L1 and 68E7 [14]. For further analyses, HPV genotypes 16, 18, 31, 33, 35, 39, 45, 51, 52, 56, 58, 59, and 68 were used as the high-risk HPV (hrHPV) genotypes. Real-time quantitative PCR for β-globin was always performed and was used as a proxy for the quality of sampling. The amount of β-globin DNA (in nanograms) present in each sample was divided by the weight of 1 genome equivalent (i.e., 6.6 picogram/cell) to obtain the number of genome equivalents in the sample.

### Statistics

Data analysis included descriptive statistics, cross-tabulations, and graphical representations using SPSS, version 22. Forest plots were created in Excel, version 2013. Odds Ratios (OR) of this nested case-control study are calculated by conditional logistic regression using COXREG in SPSS. All statistical tests were conducted at a 5 % significance level, and 95 % confidence intervals were computed.

## Results

A total of 2106 samples from FSWs between June 2006 and June 2010 were tested. Of these, 753 were excluded because they were samples from FSWs who had already been tested earlier in the study period. In addition, 3 FSWs were excluded due to HIV positivity, and 15 were excluded because age was not available. The 1334 controls were selected from 200,736 data sets from women living in the Antwerp region. The analysis was performed on samples from 1334 FSWs and 1334 age-matched controls.

### Socio-demographics

**Fig. 1:**
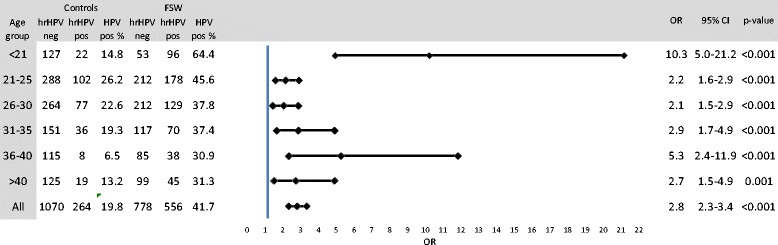
Prevalence of hrHPV DNA in female sex workers (FSWs) compared to an age-matched control group in Antwerp, Belgium

The average age of the FSWs was 29.10 years (range, 17 to 70 years). The number of FSWs in each age group is shown in Fig. 1. For each FSW, a control of the same age who was tested on average 0.88 days after the FSW was identified (618 controls were tested on the same day and 464 on the following day, and the maximum interval between testing the FSW and the control was 7 days).

Nationality was known for 1,254 FSWs (94 %). The majority (70 %, 877/1254) were European. Table 1 shows the top ten countries of origin and the mean age of the FSWs. Briefly, the top four countries of origin were Belgium, Nigeria, Bulgaria, and Romania, with 344, 130, 123, and 105 FSWs, respectively, followed by the Netherlands, no country reported, Thailand, the Dominican Republic, Poland, and Albania, with 97, 80, 75, 38, 36, and 28 FSWs, respectively. Significant differences in the average age for different nationalities were observed. The youngest FSWs were Romanian, Bulgarian and Nigerian, with an average age of 23, 24, and 26 years, respectively. Older FSWs originated from Belgium, the Dominican Republic and Thailand, with an average age of 32, 33, and 34 years, respectively. The controls were randomly selected from women living in the same region (Antwerp province). Data on nationality or origin was not available for the controls.

**Table 1:** Female sex workers top ten countries of origin and mean age

Country	Number of FSW	Mean age in years (5^th^-95^th^ percentile)
Belgium	344	32 (20–48)
Nigeria	130	26 (21–32)
Bulgaria	123	24 (18–27)
Romania	105	23 (19–28)
Netherlands	97	30 (20–46)
no country reported	80	27 (18–45)
Thailand	75	34 (21–46)
Dominical Republic	38	33 (20–48)
Poland	36	28 (19–42)
Albania	28	29 (23–35)

### hrHPV prevalence

**Fig. 2:**
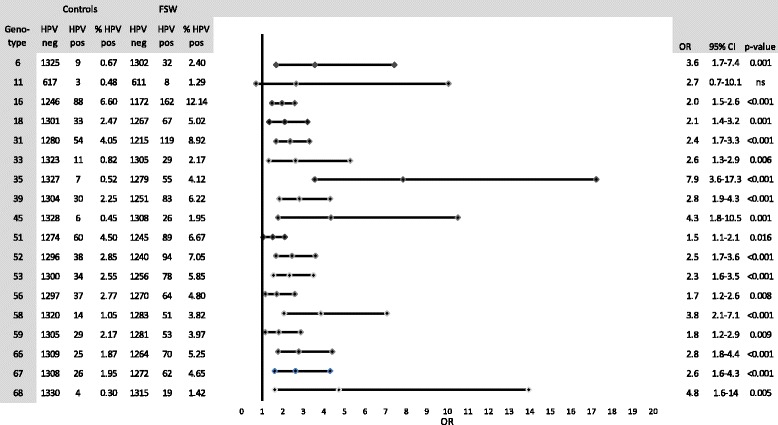
HPV genotype-specific prevalence in female sex workers (FSWs) compared to an age-matched control group in Antwerp, Belgium

The overall hrHPV prevalence for FSWs (41.7 %) was significantly higher than for the control group (19.8 %) (*p* <0.00001). The OR for hrHPV in FSWs compared to the controls was 2.8 (95 % CI: 2.3–3.4). Figure 2 provides an overview of the different HPV genotypes among FSWs and controls.

The most common hrHPV genotypes in all FSWs were HPV-16 (12.1 %), HPV-31 (8.9 %) and HPV-52 (7.0 %). The most common hrHPV genotypes in the controls were HPV-16 (6.6 %), HPV-51 (4.5 %) and HPV-31 (4.0 %).

Both groups (FSW and controls) had mostly single infections (59.3 and 65.2 %, respectively) and duo-infections (24.8 and 20.5 %, respectively). Small percentages of both groups (FSW and controls) had 3 or more hrHPV infections (15.8 and 14.4 %, respectively). There was no significant difference (*p* = 0.11) in the occurrence of multiple infections (2 or more) between groups, with 40.6 % in the FSW group and 34.8 % in the control group. Only FSWs younger than 21 years of age had significantly more multiple infections (2 or more) than did the controls, at 51.0 and 27.2 %, respectively (*p* = 0.04).

### Demographic characteristics in relation to hrHPV occurrence

Figure 1 compares the hrHPV prevalence by age in both groups. Significant differences were observed in all age groups and in particular in the age cohort under 21 years (*n* = 149) (*p* < 0.05). In FSWs younger than 21 years old, a prevalence of 64.4 % was observed with an OR of 10.3 (95 % CI: 5.0–21.2). The group between 36 and 40 years of age showed a significantly higher hrHPV prevalence among FSWs than controls, with an OR of 5.3 (95 % CI: 2.4–11.9).

### Cervical lesions (cellular abnormalities)

**Fig. 3:**
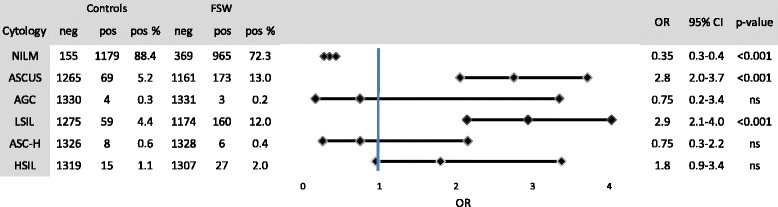
Cytology in female sex workers (FSWs) compared to an age-matched control group in Antwerp, Belgium

Figure 3 and Table 2 show the cytological results for the FSW and control groups. Cellular abnormalities were observed in approximately one-third of FSWs (27.7 %), which was significantly higher than in the controls (11.6 %) (*p* <0.05). In addition, significantly more ASCUS (atypical squamous cells of undetermined significance) and LSIL (low-grade squamous intraepithelial lesions) were diagnosed in FSWs than in controls, with ORs of 2.8 (95 % CI: 2.0–3.7) and 2.9 (95 % CI: 2.2–4.0), respectively.

**Table 2:** Cytological results of FSWs compared to age-matched controls according to different age groups

Age groups		NILM	ASC-US	AGC	LSIL	ASC-H	HSIL	Total	*p*-value*
<21	Controls	136	8		5		0	149	<0.001
	FSWs	80	36		32		1	149	
21–25	Controls	333	25	1	25	2	4	390	<0.001
	FSWs	272	50	0	57	2	9	390	
26–30	Controls	297	16	0	17	2	9	341	0.022
	FSWs	275	34	1	24	1	6	341	
31–35	Controls	166	7	0	8	4	2	187	<0.001
	FSWs	134	21	2	22	3	5	187	
36–40	Controls	115	6		2		0	123	<0.001
	FSWs	92	17		11		3	123	
>40	Controls	132	7	3	2		0	144	0.004
	FSWs	112	15	0	14		3	144	
Total	Controls	1179	69	4	59	8	15	1334	<0.001
	FSWs	965	173	3	160	6	27	1334	

*AGC* atypical glandular cells, *ASC-H* atypical squamous cells cannot exclude high-grade squamous intraepithelial lesions, *ASCUS* atypical squamous cells of undetermined significance, *HSIL* high-grade squamous intraepithelial lesions, *LSIL* low-grade squamous intraepithelial lesions

*: *p*-value is calculated using Chi Square based on cased of NILM, ASC-US and combined categories of AGC, LSIL, ASC-H and HSIL

In FSWs with AGC (atypical glandular cells) (*n* = 3), ASC-H (atypical squamous cells cannot exclude high-grade squamous intraepithelial lesions) (*n* = 6) or HSIL (high-grade squamous intraepithelial) (*n* = 27) was detected. In cases with LSIL and ASCUS, we found hrHPV prevalences of 91 % and 83 %, respectively. The hrHPV prevalence in FSWs with normal cytology was 26 %. Looking at the different age groups in table 2, a significant difference in the number of cellular abnormalities between FSWs and controls was observed in all age groups. Again, the difference was most pronounced in the youngest age group, < 21 years (*p* <0.001).

## Discussion

To our knowledge, this is the largest nested case-control study (2 N = 2668) describing hrHPV genotyping in FSWs [15]. Overall, the hrHPV prevalence was significantly higher in FSWs (41.7 %) than in the general population (19.8 %).

A 2013 literature review and analysis of the results of 35 HPV-prevalence studies in FSWs reported a median overall prevalence of HPV infections in FSWs of 42.7 %, with a range of 2.3 to 100 % [15]. This is comparable to our results (41.7 %). A 2004 study in the Belgian region of Ghent reported an HPV prevalence of 77 % in 93 FSWs [16]. However, the sample size was smaller, the detection technique was different and the population characteristics may not have been the same as in our study. Also in 2004, a study in the Antwerp region compared a group of FSWs (*N* = 61) with the general population (*N* = 84) and found that the HPV prevalence in FSWs was significantly higher (34.4 % in FSWs and 22.8 % in controls) [17].

In both groups (Fig. 2), the most common hrHPV genotype was HPV-16, and it was significantly more prevalent in the FSW (12.1 %) than in the control group (6.6 %). The second and third most common hrHPV genotypes in FSWs were HPV-31 (8.9 %) and HPV-52 (7.1 %). Although the overall prevalence of hrHPV in a review of 35 studies looking at HPV in FSWs is higher than in our results, we identified the same predominant genotypes, including HPV 16 (38.9 %), HPV 31 (28.4 %) and HPV52 (32.7 %) [15].

The decreasing prevalence of hrHPV with age that we observed in the control group confirms what is found in the literature [8, 18, 19]. It is clear that a young age also contributes to a high hrHPV prevalence, and this was equally valid for FSWs. A study in Mombasa reported an HPV prevalence of 52.1 % in 17- to 24-year-old HIV-negative FSWs, which is comparable to the 50.9 % hrHPV prevalence we found in the under 25 years cohort in this study [20].

In addition to frequent exposure to multiple sex partners, we want to emphasize the frequent use of vaginal douching in this group. This procedure is known to alter the vaginal microbiome and to frequently cause bacterial vaginosis (BV) [21]. Based on a meta-analysis of 12 studies, an association between BV and cervical HPV infection has been reported [22]. Interestingly, it has recently been shown that the conditions that prevent BV (i.e., when the vaginal microbiota is dominated by *Lactobacillus* gasseri) are associated with an increased clearance of detectable HPV [23]. The often-deregulated vaginal microbiota in FSWs may therefore also contribute to the major differences we observed between FSWs and the control group. Further studies are needed to confirm this.

### The need for tailored interventions: cervical screening

Most HPV studies in FSWs describe hrHPV prevalence, but only a few papers also provide cytological or histological results. Two studies reporting on both cytology and histology in FSWs found more cellular abnormalities in FSWs than in the general population, which confirms our findings [16, 24]. In addition, we have reported and confirmed significantly more HPV infections and abnormal smear results in the youngest age cohort (17 to 21 years old). Recent recommendations from the Belgian Health Care Knowledge Centre advise starting cytology screening at the age of 25 with an interval of 3 years until age 30 and then performing HPV DNA screening every 5 years [25]. However, after observing the significant differences in hrHPV prevalence and abnormal cytology between the FSW and control groups, it is clear that further research is needed to protect FSWs from the consequences of HPV infections. In addition, targeting FSWs is challenging as they tend to move within and between countries, and an effective screening follow-up may be difficult if the time interval between two samplings is too long. These observations support the need for further research on tailored screening guidelines. Starting earlier, sampling more frequently and referring FSWs for colposcopies sooner are all options to be considered.

### The need for tailored interventions: HPV vaccination

Regarding HPV vaccination, the current guidelines for older women may need to be refined. It has been clearly demonstrated that HPV vaccines are most effective when administered before exposure to HPV because they have no therapeutic effects and protect against HPV types not yet acquired at the time of vaccination [26, 27]. For the general population, it has been calculated that the vaccination of women up to age 30 may still be cost effective [28]. At later ages, the cost effectiveness decreases. This is partly due to limited new infections in women over age 30. However, the career of an FSW can last a lifetime (the results presented here are taken from FSWs between 17 and 70 years old), and as frequent sexual contact with ‘non-vaccinated’ males from the general population may be substantial, exposure to hrHPV continues. In addition, we know that some women start sex work at a later age. Indeed, in this study, we also report a significant difference in hrHPV DNA positivity in 31- to 40-year-old FSWs compared to the controls (*p* < 0.0001).

The health benefits of vaccination in this high-risk group, which has significantly more HPV infections, more cervical lesions and more exposure to non-vaccinated males, may be substantially different than in women from the general population. It seems obvious to investigate, in line with other occupational health immunisations, whether it would be beneficial to offer catch-up vaccinations for this specific at risk population.

In Flanders, free vaccinations for one age-cohort of girls between 10 and 13 years of age have been offered since 2010 through a school vaccination programme. In addition, a free catch-up vaccine is offered up to age 18. In the first year of implementation (the 2010–2011 school year), 3 doses of the vaccine were administered to 83.5 % of the 11 year old girl population [29]. This means that the majority of Flemish FSWs may soon have received the HPV vaccination. However, such a preventative programme covers only part of the FSW population because only 25 % of the FSWs in this study were Belgian. In addition, lower vaccination coverage in Flanders was linked to lower social class [30]. Therefore, the impact of the national vaccination programme may be lower in this high-risk group. This justifies additional research on the potential benefits and feasibility of catch-up vaccinations in the FSW population.

### Strengths and limitations

To our knowledge, this is the largest study on HPV genotyping in FSWs. The city of Antwerp has 510,000 inhabitants representing more than 160 different nationalities as well as a major port. This diversity is also reflected in the population of FSWs visiting the health house (http://www.ghapro.be/en/index.html), where 65 different nationalities are registered. We only included FSWs who came for routine screening for cervical cancer and not on FSWs in general, although the test is available if necessary. Furthermore, the selection of the controls with perfect case control match for age and testing date is a unique feature of this study. A bias could have occurred only in the youngest age cohort because there are no standard screening guidelines for this cohort, and we may have selected proportionally more sexually active girls. In Belgium, a number of treating physicians and gynaecologists do screen young girls (<21 years) during consultations for oral contraceptive prescriptions. In addition, many physicians screen young girls after childbirth. In both cases, these women were sexually active, and any selection bias would generate a higher prevalence in the youngest control group and potentially mask the specific impact of sex work at young ages. A number of physicians and gynaecologists routinely take pap smears starting at the age of 21, and this should be a representative control group. These data clearly confirm the previously reported increased hrHPV prevalence and increased number of abnormal cervical smears in this high-risk population.

Although we selected cases and controls that were reported for the first time in the database, we cannot exclude the possibility that both cases and controls were previously tested by another laboratory or in their country of origin. However, in most countries, including Belgium, no official screening programme is available for young women, so we expect that this had a limited impact on the reported prevalence. Another potential bias in this study is the sampling. The samples from the control group contained significantly more human DNA than did the FSW samples collected by Ghapro (data not shown). It is not clear whether this is due to possible differences in the level in the cervix from multiple washings, the application of creams, or other factors. However, the collected samples still contained enough material to adequately detect an HPV infection. Even if this influenced the presented results, it would have led to an underestimation of HPV infection in FSWs, again masking the impact of sex work on HPV prevalence and abnormal cytology.

## Conclusions

This study confirms that FSWs have an increased hrHPV prevalence as well as an increased prevalence of premalignant lesions. This effect is very pronounced in the under 21 years group. To protect this higher-risk population, specific prevention and control measures may be appropriate, including adapted HPV screening and vaccination programmes. Tailored prevention and control interventions that take into account the multiple occupational contacts of FSWs will also lead to a direct benefit for the general population.

## Abbreviations

AGC: atypical glandular cells
ASC-H: atypical squamous cells cannot exclude high-grade squamous intraepithelial lesions
ASCUS: atypical squamous cells of undetermined significance
HPV: human papillomavirus
hrHPV: high-risk human papillomavirus
HSIL: high-grade squamous intraepithelial lesions
LSIL: low-grade squamous intraepithelial lesions
PAP: Papanicolaou
PCR: polymerase chain reaction
STI: sexually transmitted infections
